# Elevated C-reactive protein in early COVID-19 predicts worse survival among hospitalized geriatric patients

**DOI:** 10.1371/journal.pone.0256931

**Published:** 2021-09-10

**Authors:** Adeline Villoteau, Marine Asfar, Marie Otekpo, Jocelyne Loison, Jennifer Gautier, Cédric Annweiler, on behalf of the GERIA-COVID study group

**Affiliations:** 1 School of Medicine, Health Faculty, University of Angers, Angers, France; 2 Department of Geriatric Medicine and Memory Clinic, Research Center on Autonomy and Longevity, University Hospital, Angers, France; 3 UPRES EA 4638, University of Angers, Angers, France; 4 Gérontopôle Autonomie Longévité des Pays de la Loire, Pays de la Loire, France; 5 Robarts Research Institute, Department of Medical Biophysics, Schulich School of Medicine and Dentistry, The University of Western Ontario, London, ON, Canada; Cleveland Clinic, UNITED STATES

## Abstract

**Background:**

The objective of this cohort study was to determine whether elevated CRP in early COVID-19 was associated with 14-day mortality in geriatric patients.

**Methods:**

Plasma CRP levels at hospital admission and 14-day all-cause mortality were assessed in geriatric inpatients hospitalized for COVID-19. Potential confounders were age, sex, functional abilities, history of malignancies, hypertension, cardiomyopathy, albuminemia, number of acute health issues, use of antibiotics and respiratory treatments.

**Results:**

Ninety-five participants (mean±SD 88.0±5.5years; 49.5%women; mean CRP, 76.7±77.5mg/L; mean albuminemia, 32.9±6.0g/L) were included. Sixteen participants who did not survive at day 14 exhibited higher CRP level at baseline than the others (120.3±71.2 versus 67.9±76.1 mg/L, P = 0.002). There was no difference in albuminemia (P = 0.329). Plasma CRP level was directly associated with 14-day mortality (fully adjusted HR = 1.11, P = 0.025). The cut-off for CRP associated with 14-day mortality was set at 35mg/L (sensitivity = 0.88; specificity = 0.56). Those with CRP<35mg/L had longer survival time than the others (log-rank P<0.001).

**Conclusions:**

Elevated CRP levels were associated with poorer 14-day survival in hospitalized geriatric COVID-19 patients.

## Introduction

The discovery of SARS-CoV-2 in Wuhan, China, at the end of 2019 and the subsequent global COVID-19 pandemic poses an unprecedented challenge for modern society. The pandemic results, particularly among older adults with comorbidities, in a high mortality rate mainly due to a poorly controlled inflammatory cascade reaction called cytokine storm and subsequent acute respiratory distress syndrome (ARDS) [1–3].

How SARS-CoV-2 causes such a cytokine storm mostly in older adults is not fully elucidated [4]. Aging is associated with chronic low-grade inflammation (aka inflammaging) characterized by higher local and systemic pro-inflammatory cytokine levels associated with a predominantly atypical ARDS [5–7]. C-reactive protein (CRP), an acute phase glycoprotein produced by the liver in response to interleukin 6 (IL-6), is a useful marker of inflammaging commonly used in clinical practice [8], as well as a sign of serious bacterial infection, trauma and various chronic diseases widely met in older adults such as malignancies for example. Although CRP potently predicts adverse outcome in these diseases, experimental data suggest that CRP may instead play a protective role in alveolitis and previous clinical evidence reported that elevated levels of CRP were associated with decreased mortality among patients with acute lung injury [9, 10]. There was also some evidence that decreased albumin levels, another strong prognostic indicator of inflammation, may be of greater value than CRP in predicting and monitoring the severity and course of ARDS in critically patients with or at risk for the syndrome after new onset fever [11, 12]. Therefore, it remains unclear whether the elevation of CRP in the early stages of COVID-19 could serve as a prognostic marker in patients with COVID-19, especially in the group of older adults. The aim of the present longitudinal cohort study was to determine whether elevated CRP levels measured in the early stages of COVID-19 were associated, independently from the albumin levels, with higher 14-day mortality in geriatric patients hospitalized for COVID-19.

## Materials and methods

The GERIA-COVID study is a longitudinal observational study conducted in the geriatric acute care unit dedicated to COVID-19 patients in the University Hospital of Angers, France, during the first wave of the COVID-19 pandemic (ClinicalTrials.gov NCT04560608). Data of the GERIA-COVID study were retrospectively collected from patients’ records. The detailed procedure has been previously described elsewhere [13].

### Study population

The inclusion criteria were the following: i) patients aged 75 years and over admitted into the geriatric acute care unit of the University Hospital of Angers, France, during the first wave of the pandemic (between March and June 2020); ii) no objection from the patients and/or their relatives for using anonymized clinical and biological data for research purpose; iii) diagnosis of COVID-19 with RT-PCR and/or chest CT-scan; iv) data available on the plasma CRP and albumin levels at hospital admission; v) data available on the vital status 14 days after the diagnosis of COVID-19.

### Plasma level of C-reactive protein

All patients had a blood test on the day of admission into hospital, along with the diagnostic tests for COVID-19. Plasma CRP levels were measured in mg/L locally at the laboratory of the University Hospital of Angers, France, with latex-enhanced immunoturbidimetry assay (ADVIA Chemistry System, Bayer Healthcare AG, Leverkusen, Germany). Elevated plasma CRP levels were defined using a threshold value calculated from a sensitivity-specificity analysis to best fit the studied group.

### Overall mortality

The main outcome was all-cause mortality at day 14. Follow-up started from the day of COVID-19 diagnosis for each patient and continued for 14 days or until death when applicable. This follow-up was long enough to cover the critical period of the 7 to 10 days of COVID-19 during which the cytokine storm may occur with its severe clinical consequences such as the often-fatal ARDS.

### Covariables

Potential confounders were age, female sex, functional abilities, history of malignancies, history of hypertension, history of cardiomyopathy, albuminemia, lymphopenia, number of acute health issues at hospital admission, use of antibiotics and use of pharmacological treatments for respiratory disorders.

Functional abilities prior to COVID-19 were measured from 1 to 6 (best) with the Iso-Resources Groups (GIR) [14]. Plasma lymphocytes (in G/L) and albumin concentration (in g/L) were measured on admission with standard techniques locally at the University Hospital of Angers, France. Lymphopenia was defined as lymphocytes ≤1G/L. Acute health issues were defined as diseases with sudden onset and rapid progression, whatever their nature or site [15]. History of hematological and solid malignancies, of hypertension and of cardiomyopathy were noted from the medical register, and by interviewing patients, their relatives and family physicians. The use of antibiotics (i.e., quinolones, beta-lactams, sulfonamides, macrolides, lincosamides, aminoglycosides, among others) and/or pharmacological treatments for respiratory disorders (i.e., beta2-adrenergic agonists, inhaled corticosteroids, antihistamines, among others) were noted from prescriptions during hospitalization.

### Statistical analysis

The participants’ characteristics were summarized using means and standard deviations (SD) or frequencies and percentages, as appropriate. As the number of observations was higher than 40, comparisons were not affected by the shape of the error distribution and no transformation was applied [16]. Firstly, comparisons between participants separated according to the vital status 14 days after the diagnosis of COVID-19 were performed using Chi-square test (or Fisher exact test) or Student *t* test (or Mann-Whitney Wilcoxon test according to the normality assessment), as appropriate. Secondly, a multiple Cox regression was used to examine the associations of 14-day mortality (dependent variable) with plasma CRP level and covariables (independent variables). The model produces a survival function that provides the probability of death at a given time for the characteristics supplied for the independent variables. Thirdly, the cut-off value for plasma CRP associated with 14-day survival was determined by a sensitivity analysis. Finally, the elapsed time to death was studied by survival curves computed according to Kaplan-Meier method and compared by log-rank test. P-values<0.05 were considered significant. All statistics were performed using SAS® version 9.4 software (Sas Institute Inc) and R (R core Team, 2018).

### Ethics

The study was conducted in accordance with the ethical standards set forth in the Helsinki Declaration (1983). All data were fully anonymized in this retrospective study of medical records. The Ethics Board of the University Hospital of Angers, France, approved the study and waived the requirement for informed consent (2020/100). No participant or relatives objected to the use of anonymized clinical and biological data for research purposes. The study protocol was also declared to the National Commission for Information Technology and civil Liberties (CNIL; ar20-0087v0).

## Results

Ninety-seven patients were consecutively diagnosed in the unit with COVID-19 during the study period and were recruited in the GERIA-COVID cohort study. Among them, two participants had missing values of plasma CRP and albumin levels. The vital status of all participants was known at day 14. Ninety-five participants were finally included in the present analysis (mean±SD, 88.0±5.5 years; 49.5% women; mean CRP level, 76.7±77.5 mg/ L; mean albumin concentration, 32.9±6.0 g/L).

Table 1 indicates the characteristics of the participants who survived at day 14 (n = 79) or those who did not (n = 16). The two groups were similar at baseline, except for the proportion of participants with a history of malignancies (Table 1). The plasma CRP level was higher in those who did not survive at day 14 (respectively, 120.3±71.2 versus 67.9±76.1 mg/L, P = 0.002). There was no difference in albumin level (P = 0.329).

**Table 1:** Characteristics and comparison of COVID-19 geriatric patients separated into two groups according to 14-day mortality (n = 95).

	Total cohort (n = 95)	14-day mortality		P-value*
		No (n = 79)	Yes (n = 16)	
**Demographical data**				
Age (years), mean±SD	88.0±5.5	87.8±5.6	89.2±4.8	0.353
Female sex	47 (49.5)	39 (49.4)	8 (50.0)	0.963
GIR score (/6), mean±SD	3.5±1.5	3.7±1.4	2.9±1.7	0.051
**Comorbidities**				
Hematological and solid malignancies	32 (33.7)	22 (26.6)	11 (68.8)	**0.001**
Hypertension	60 (63.2)	49 (62.0)	11 (68.8)	0.611
Cardiomyopathy	50 (52.6)	40 (50.6)	10 (62.5)	0.386
**Hospitalization**				
Plasma CRP (mg/L), mean±SD	76.7±77.5	67.9±76.1	120.3±71.2	**0.002**
Plasma albumin (g/L), mean±SD	32.9±6.0	33.2±6.1	31.6±5.7	0.329
Lymphopenia	42 (4.2)	34 (43.0)	8 (50.0)	0.609
Number of acute health issues at hospital admission, mean±SD	2.9±1.6	2.8±1.6	3.3±1.5	0.317
Use of antibiotics^‡^	64 (67.4)	51 (64.6)	13 (81.3)	0.194
Use of pharmacological treatments for respiratory disorders^||^	11 (11.6)	8 (10.1)	3 (18.8)	0.389

Data presented as n (%) where applicable; COVID-19: Coronavirus Disease 2019; CRP: C-reactive protein; GIR: Iso Resource Groups

*: between-group comparisons based on Chi-square test (or Fisher exact test) or Student *t* test (or Mann-Whitney Wilcoxon test according to the normality assessment), as appropriate

‡: quinolones, beta-lactams, sulfonamides, macrolides, lincosamides, aminoglycosides, among others

||: beta2-adrenergic agonists, inhaled corticosteroids, antihistamines, among others.

Table 2 shows a direct association between plasma CRP level at baseline and 14-day mortality. The hazard ratio (HR) for mortality was 1.06 [95% confidence interval (CI): 1.01;1.12] (P = 0.012) in the unadjusted model, and 1.11 [95%CI: 1.02;1.21] (P = 0.014) after adjustment for all potential confounders. The history of malignancies was also associated with greater mortality risk (HR = 7.65, P = 0.001) in the adjusted model.

**Table 2:** Multiple Cox proportional-hazards model showing the hazard ratio for 14-day mortality (dependent variable) according to baseline plasma CRP (independent variable), adjusted for participants’ characteristics (n = 95).

	14-day mortality			
	Unadjusted model		Fully-adjusted model	
	HR [95% CI]	P-value	HR [95% CI]	P-value
Plasma CRP level (for 10mg/L)	1.06 [1.01; 1.12]	**0.012**	1.11 [1.02; 1.21]	**0.014**
Age	1.05 [0.95; 1.15]	0.354	1.01 [0.89; 1.15]	0.859
Female sex	1.00 [0.38; 2.67]	0.995	1.58 [0.42; 5.94]	0.500
GIR score	0.71 [0.50; 1.02]	0.061	0.80 [0.51; 1.27]	0.342
Hematological and solid malignancies	4.84 [1.68; 13.95]	**0.004**	7.65 [2.27; 25.76]	**0.001**
Hypertension	1.30 [0.45; 3.75]	0.622	1.94 [0.54; 7.02]	0.310
Cardiomyopathy	1.52 [0.55; 4.18]	0.419	1.16 [0.34; 3.93]	0.809
Plasma albumin concentration	0.96 [0.88; 1.04]	0.301	1.07 [0.95; 1.21]	0.240
Lymphopenia	1.26 [0.47; 3.37]	0.640	1.01 [0.27; 3.79]	0.989
Number of acute health issues at hospital admission	1.17 [0.86; 1.60]	0.273	1.11 [0.71; 1.72]	0.648
Use of antibiotics^†^	2.16 [0.62; 7.58]	0.230	2.67 [0.54; 12.23]	0.237
Use of pharmacological treatments for respiratory disorders^‡^	1.94 [0.55; 6.81]	0.301	2.44[0.46; 12.89]	0.295

CI: confidence interval; COVID-19: coronavirus disease 2019; CRP: C-reactive protein; GIR: Iso Resource Groups; HR: hazard ratio

†: quinolones, beta-lactams, sulfonamides, macrolides, lincosamides, aminoglycosides, among others

‡: beta2-adrenergic agonists, inhaled corticosteroids, antihistamines, among others.

**Fig 1:**
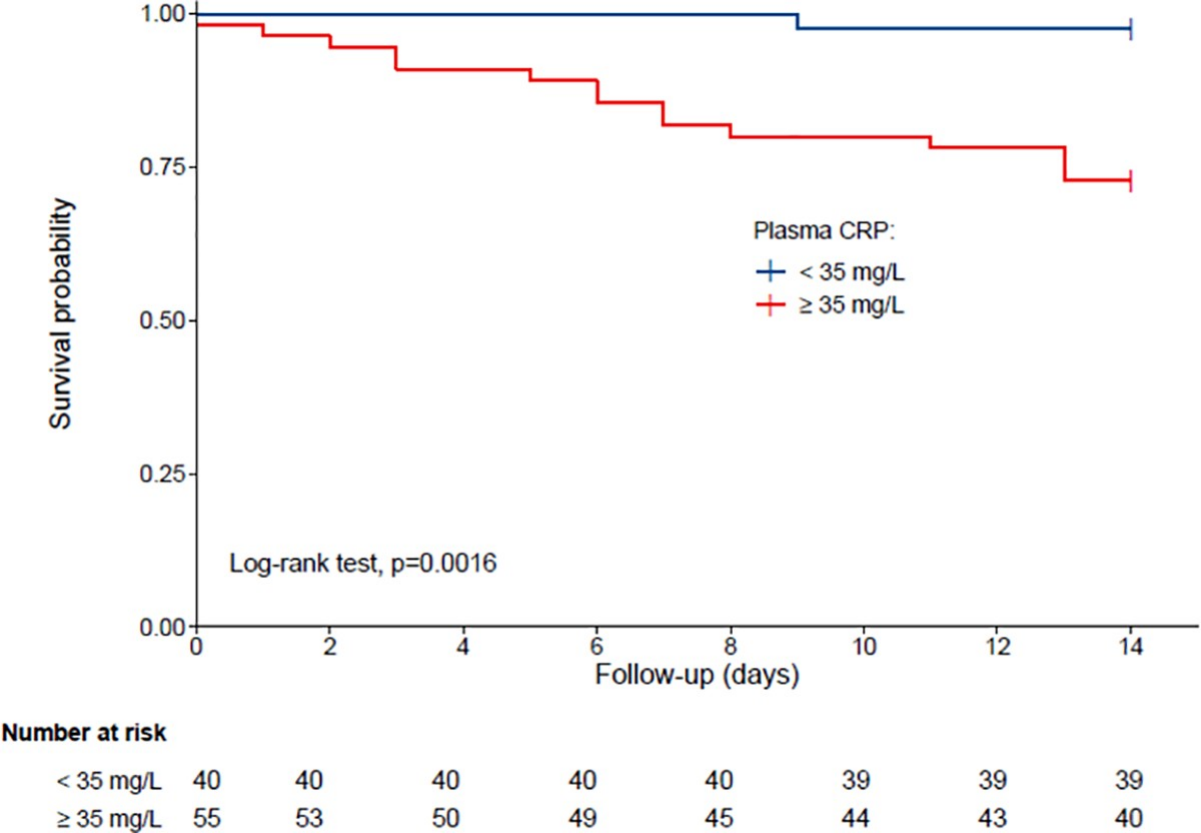
Kaplan-Meier estimates of the cumulative probability of COVID-19 participants’ survival according to plasma CRP (n = 95). Group 1: plasma CRP < 35 mg/L; Group 2: plasma CRP ≥ 35 mg/L.

The cut-off value for plasma CRP associated with mortality was set at 35 mg/L, as determined by the sensitivity analysis (sensitivity = 0.88; specificity = 0.56). Kaplan-Meier distributions showed in Fig 1 that COVID-19 participants with CRP<35 mg/L had longer survival time than the others (log-rank P = 0.0016).

## Discussion

The main result of this cohort study is that, irrespective of all measured potential confounders including albuminemia, the elevated CRP levels in early COVID-19 were associated with higher 14-day mortality among geriatric patients with COVID-19. This novel finding encourages the understanding of CRP as a possible prognostic marker in COVID-19 even in older adults. Further studies are needed to determine whether drugs aimed at normalizing CRP levels may improve COVID-19 survival.

The association between CRP levels and COVID-19 prognosis has been the subject of some previous studies [17]. Bannaga et al. reported in 321 adults with COVID-19 that increased CRP and decreased albumin levels on admission to intensive care units were associated with greater mortality after adjustment for advancing age [18]. Similarly, higher levels and a higher velocity of increase of CRP levels were observed in non-survivors compared to survivors among 577 middle-aged adults hospitalized for COVID-19 [19]. Consistently, the CRP level was also found as one of the variables most predictive of respiratory failure and of the need for mechanical ventilation in 89 adults hospitalized for COVID-19 [20]. Compared to our analysis, these previous studies did not focus on older adults specifically. Our study therefore provides novel information by confirming that the elevation of CRP above 35 g/L predicts fatal outcomes in COVID-19 also in older adults despite the inflammaging that usually characterizes this population. Confirming such link in COVID-19 is even more important since this result is contrary to what was previously reported in non-COVID-19 ARDS, in which the increase in CRP is thought to be protective instead [10].

How CRP is associated with decreased survival in older adults with COVID-19 is not yet fully understood. First, CRP in the early stage of COVID-19 has been reported to be positively correlated with lung lesions [21, 22] and may thus be a biomarker of the disease severity [23, 24]. Second, since the synthesis of CRP by hepatic cells is linked to IL-6, the levels of CRP might reflect the IL-6 secretion due to the activation by SARS-CoV-2of monocytes, macrophages, and dendritic cells. The clinical importance is linked to the fact that IL-6 is involved in the cytokine storm, which subsequently involves the secretion of VEGF and a decrease in the expression of E-cadherin contributing to greater vascular permeability, arterial hypotension, organ failures and ARDS, with the risk of fatal outcome [25]. Third, the inflammatory state illustrated by the elevation of CRP could induce a prothrombin state with consequent increased risks of arterial events such as stroke [26] or venous thromboembolic events [27] such as pulmonary embolism [28]. Fourth, elevated CRP may also be associated with higher mortality risk by inducing hypercatabolism consuming respiratory muscle proteins with subsequent less opportunity for compensatory responses in respiratory distress. Fifth, the level of CRP may be the marker of the pre-COVID-19 health status of older individuals and illustrates both the burden of chronic diseases -which are mostly risk factors for severe COVID-19- and the inflammaging. Inflammaging is the chronic activation of innate immune system while advancing in age, which results in a low-grade, chronic, controlled inflammation in older adults [29, 30]. Inflammaging is represented by increased number of natural killer cells and increased production of pro-inflammatory cytokines, especially IL-6 and CRP [31]. This chronic inflamed state has detrimental effect on health, contributes to biological aging, and may explain the frequency of severe and fatal forms of COVID-19 in the elderly population.

The finding that higher CRP levels are associated with higher mortality risk in older patients with COVID-19 has interesting potential clinical and research implications. To the best of our knowledge, there are no clear reference on what a ‘pathological’ CRP value is in the early stage of COVID-19 among older adults. Such estimates of a cut-off value around 35 mg/L may help to justify, plan, evaluate, and compare the efficacy of interventions aimed at improving COVID-19 survival based on the monitoring and/or correction of inflammation in older adults with COVID-19.

Some limitations should be acknowledged. First, the present study was single-centered and participants restricted to geriatric patients hospitalized for COVID-19 who might be not representative of the general population of older adults. Second, although we were able to control for important characteristics that could modify the association, residual potential confounders might still be present such as the IL-6 levels. Third, the observational design of our study was less robust than an interventional study and precluded any causal inference. Fourth, the diagnostic strategy for COVID-19 was heterogeneous, sometimes relying only on a chest CT-scan as was recommended during the first wave of the pandemic [32], although more recent studies showed that the assessment of radiological signs is highly dependent on the experience of the radiologist and not objective [33].

## Conclusions

In conclusion we found that, irrespective of potential confounders including albuminemia, elevated CRP levels measured in the early stages of COVID-19 were associated with lower 14-day survival among hospitalized geriatric patients. Biomarkers’ clinical usefulness is defined as their capability to influence clinicians to diagnose the disease, predict prognosis, and guide treatment. Here, the elevation of CRP above 35 mg/L is certainly not credible as a diagnostic marker of COVID-19 but may be considered as a useful prognostic marker for severe forms of COVID-19, even in older adults. In the context of the emergence of immune-escape SARS-CoV-2 variants, continued efforts should be made to continue exploring the value of drugs aimed at normalizing the inflammatory state, such as corticosteroids or immunosuppressants, to improve the ability of the host to resist infection and to prevent the fatal consequences of the cytokine storm.
